# 吉非替尼治疗非小细胞肺癌的*meta*分析

**DOI:** 10.3779/j.issn.1009-3419.2011.04.09

**Published:** 2011-04-20

**Authors:** 继武 郭, 彬 马, 慧银 周, 瑶 王, 圆 张

**Affiliations:** 1 730000 兰州，兰州大学循证医学中心，兰州大学基础医学院 Evidence-Based Medicine Center of Lanzhou University, School of Basic Medical Science, Lanzhou University, Lanzhou 730000, China; 2 730030 兰州，兰州大学第二临床医学院 Second Clinical Medical College of Lanzhou University, Lanzhou 730030, China

**Keywords:** 吉非替尼, 肺肿瘤, *Meta*分析, Geftinib, Lung neoplasms, *Meta* analysis

## Abstract

**背景与目的:**

非小细胞肺癌（non-small cell lung cancer, NSCLC）是恶性程度和死亡率极高的肿瘤，吉非替尼是近年来研发的一种新的分子靶向药物，本文旨在系统评价吉非替尼治疗NSCLC的有效性和安全性。

**方法:**

计算机检索Cochrane图书馆（2010年第8期）、PubMed、Embase、CNKI、VIP，中华医学会数字化期刊（截至2010年8月）。两名评价者独立评价纳入研究的质量、提取资料并交叉核对，同质研究采用RevMan 5.0软件进行*meta*分析。

**结果:**

共纳入13个随机对照试验，包括6, 207例病例。*Meta*分析结果显示吉非替尼相比较于安慰剂、多西紫杉醇、顺铂+多西紫杉醇、培美曲赛等治疗方案而言，中位生存时间、1年生存率、完全缓解率、部分缓解率、疾病无进展率等方面未显示出优势。与多西紫杉醇、顺铂+多西紫杉醇相比，吉非替尼可明显增加化疗患者的总有效率（RR=1.41, 95%CI: 1.10-1.80; RR=1.93, 95%CI: 1.26-2.94）。吉非替尼对比安慰剂、多西紫杉醇能够提高患者的生存质量和总FACT-L改善率（RR=1.42, 95%CI: 1.16-1.74; RR=1.66, 95%CI: 1.39-1.97）。吉非替尼的主要不良反应包括皮疹/痤疮、皮肤干燥、腹泻，其血液毒性较低。

**结论:**

吉非替尼治疗NSCLC有一定的优势，可作为治疗NSCLC的常规药物。

## Abstract (en)

**Background and objective:**

Malignant grade and death rate are very high for non-small cell lung cancer, and geftinib is a new molecule target anticancer drug. The aim of this *meta* analysis is to evaluate the clinical efficacy and safety of geftinib for non-small cell lung cancer.

**Methods:**

We searched the Cochrane Central Register of Controlled Trials (CENTRAL) (The Cochrane Library, Issue 8, 2010), PubMed (1966-2010.8), EMBASE (1974-2010.8), CNKI (1994-2010.8), VIP (1989-2010.8), and CMD Digital Periodicals (1998-2010.8). Two reviewers independently evaluated the quality of the included studies and extracted the data. *Meta*-analyses were performed by RevMan 5.0 sofware.

**Results:**

Tirteen randomized controlled trials (RCTs) involving 6, 207 patients were included. The results of *meta*-analyses showed that: geftinib showed no remarkable advantage in media survival time, 1 year survival rate, complete response rate (CRR), partial response rate (PRR), stable disease (SD) when compared to Placebo, Docetaxel, Cisplatin+Docetaxel, Pemetrexed. Giftinib could increase overall survival rate compared to Docetaxel, Cisplatin+Docetaxel (RR=1.41, 95%CI: 1.10-1.80; RR=1.93, 95%: 1.26-2.94). When compared to Placebo, Docetaxel, giftinib could improve life quality of Total-FACT-L improve rate (RR=1.42, 95%CI: 1.16-1.74; RR=1.66, 95%CI: 1.39-1.97). The major adverse event for giftinib were rash/acne, dry skin, diarrhea. While giftinib showed lower hematology toxicity.

**Conclusion:**

Giftinib shows more superiority for non-small cell lung cancer, and its clinical application is worthy to be advocated.

近十年全球范围内肺癌仍是癌症中导致死亡的最主要原因之一^[1-4]^。2009年我国卫生统计年鉴显示，肺癌导致的死亡高居所有癌症死亡的首位（30.83/10万），且男性明显高于女性（41.34/10万*vs*19.84/10万）^[5]^。

非小细胞肺癌（non-small cell lung cancer, NSCLC）早期通常无症状，70%-80%患者在确诊时已属晚期。含铂类的化疗药物是目前晚期NSCLC的标准一线化疗方案，但总有效率仅为30%-40%，中位生存期仅为8个月-11个月^[6]^。多西紫杉醇是二线化疗中的首选药物，可有效延长患者的中位生存时间^[7]^。近年来随着对基因和信号转导研究的进展，分子靶向治疗为肿瘤的治疗提供了新的思路^[8]^。吉非替尼（易瑞沙）是一种口服的表皮生长因子受体酪氨酸激酶抑制剂。在Ⅱ期临床试验中，对于进展期或脑转移的NSCLC口服250 mg/d吉非替尼有明显的抗肿瘤效果，且耐受性较好^[9, 10]^。但也有研究^[11, 12]^报道，吉非替尼并不能增加NSCLC患者的总生存率和无疾病生存期，且出现更多的毒性反应。

因此，本文按照Cochrane系统评价方法，对吉非替尼治疗NSCLC的临床疗效和安全性进行系统全面的评估，以期为临床应用提供科学依据。

## 1 材料与方法

### 1.1 纳入/排除标准

#### 1.1.1 研究类型

吉非替尼治疗NSCLC的随机对照试验，无论是否采用盲法。

#### 1.1.2 研究对象

① 年龄≥18岁，无性别限制；②经病理确诊为NSCLC；③Kamofsky评分≥60分或ECOG评分为0分-2分；④治疗前无化疗禁忌症，肝肾功、血液学、心电图无明显异常。排除合并有小细胞肺癌或其它恶性肿瘤、肝肾功能损害者；先前化疗方案中包含与吉非替尼机理相似的药物；未签署知情同意书者。

#### 1.1.3 干预措施

吉非替尼*vs*安慰剂或其它化疗药物（多西紫杉醇、卡铂、顺铂、紫杉醇、吉西他滨或培美曲赛等），不限制吉非替尼和其它药物的剂量和疗程。

#### 1.1.4 结局指标

主要测量指标：中位生存期（media survival）、1年生存率（1-year survival rate）；次要测量指标：完全缓解（complete response, CR）、部分缓解（partial response, PR）、疾病稳定（stable disease, SD）、总有效率（overall response rate, ORR）、疾病控制率（disease control rate, DCR）、生活质量改善率和不良反应。其中生活质量改善率包括TOI（trial outcome index）改善率、LCS（lung cancer subscale）改善率、总FACT-L（Total Functional Assessment of Cancer Terapy-Lung）改善率；不良反应包括皮疹/痤疮（rash/acne）、腹泻（diarrhea）、中性粒细胞减少（neutropenia）和贫血（anemia）。

#### 1.1.5 判效标准

生存期从病人被随机分组至不能耐受化疗或者死亡为止。ORR=（CR+PR）/总例数，DCR=（CR+PR+SD）/总例数；当TOI和总FACT-L评分高于基础评分6分时定义为改善，LCS评分高于基础评分2分时定义为改善。

### 1.2 检索策略

计算机检索Cochrane图书馆（2010年第8期）、PubMed（1966年1月-2010年8月）、EMbase（1974年1月-2010年8月）、CNKI（1994年1月-2010年8月）、VIP（1989年1月-2010年8月）。检索词包括：非小细胞肺癌、吉非替尼、易瑞沙、non-small cell lung carcinoma、NSCLC、geftinib和iressa等。

### 1.3 资料提取和质量评价

由2位研究者按照设计好的资料提取表，独立提取资料并交叉核对，保证数据的准确性。随机对照试验的质量评价方法，采用Cochrane Handbook 5.0.2的标准^[13]^，包括随机方法、隐蔽分组方法、盲法、不完整数据报告、选择性报告研究结果和其它偏倚来源6方面。

### 1.4 资料分析

统计学分析采用Cochrane协作网提供的RevMan 5.0版统计软件。计数资料选择优势比（RR）作为效应尺度指标，连续变量选用加权均数差（MD）作为统计效应量，同时计算95%的可信区间（CI）。RR > 1和MD > 0时表示试验组的效应量大于对照组，RR < 1时表示试验组效应量小于对照组。假设检验采用卡方检验，*P* < 0.05为有统计学差异。当纳入研究统计学异质性检验结果为*P* > 0.1，*I^2^* < 50%，采用固定效应模型，反之采用随机效应模型。

## 2 结果

### 2.1 检索结果和纳入研究的一般特征（表 1）

**1:** 纳入研究特征 Character of included studies

Study	Intervention	Participant	Median age (Range)	No. of center	Place of trial	Scheme of chemotherapy	Endpoint
Giuseppe Giaccone 2004^[14]^	gemcitabine+cisplatin+gefitinib	365 (280/85)	59(34-83)	Multi-center (155)	Europe、North American、Asia、South America	Gefitinib or placebo was administered orally, once daily. Chemotherapy was administered in 3-week cycles for a total of six cycles：intravenous gemcitabine 1, 250 mg/m^2^ for 30 minutes on days 1 and 8; intravenous cisplatin 80 mg/m^2^ after gemcitabine administration on day 1 only. Subsequently, patients continued on gefitinib or placebo until disease progression.	Mediansurvivaltime, 1 year survival rate, tumor responses, safety, diarrhea, rash/acne, Hematologictoxicity
	gemcitabine+cisplatin+ placebo	363 (262/101)	61 (33-81)				
RoyS. Herbst 2004^[15]^	paclitaxel+ carboplatin+ gefitinib	345 (199/146)	61 (27-86)	Single-center	USA	All patients received chemotherapy (intravenous paditaxel 225 mg/m^2^ over 3 hours on day 1 of a 3-weekcyde immediately followed by intravenous carboplatin area under concentration/time curve of 6 mg/min/mLover 15 to 30 minutes on day 1) and oral gefitinib at 250 mg/d or daily oral placebo. Chemotherapy was continued for six cycles in the absence of disease progression.	Survival, tumor responses, quality of life, vomiting, diarrhea, rash/ acne, Hematologictoxicity
	paclitaxel+ carboplatin+ placebo	345 (212/133)	63 (31-85)				
Nick Thatcher 2005^[16]^	gefitinib	1, 129(761/368)	62 (28-90)	Multi-center (210)	Europe, Asia, South America, Australia, Canada	gefitinib (250 mg/d) or placebo until unacceptable toxic effects occurred, consent was withdrawn, or the patient was no longer deriving clinical benefit.	Median survival time, 1 year survival rate, tumor responses, rash/acne, vomiting, diarrhea, life quality
	placebo	563 (378/185)	61 (31-87)				
XIONG Huihua 2008^[17]^^a^	gefitinib			Single-center	China	Gefitinib 250 mg/m^2^ was administered orally with 30 min, docetaxel was administered every 3 weeks as a 1-hour intravenous infusion of 75 mg/m^2^.	Hematologictoxicity, rash/acne, diarrhea
	docetaxel						
Riichiroh Maruyama 2008^[12]^	gefitinib	245 (151/94)	—	Single-center	Japan	Gefitinib 250 mg/d was administered orally; docetaxel was administered every 3 weeks as a 1-hour intravenous infusion of 60 mg/m^2^. Patients received treatment until disease progression, intolerable toxicity, or discontinuation for another reason.	Progression-free survival.
	docetaxel	244 (151/93)	—				ORR, DCR, qualityof life
Edward S Kim 2008^[18]^	gefitinib	733(466/267)	61 (27-84)	Multi-center (149)	Europe, Asia, South America	gefitinib (250 mg/d orally) or docetaxel (75 mg/m^2^ in a 1-hour infusion every 3 weeks) until disease progression, unacceptable toxic effects, or patient or physician request to discontinue treatment. The docetaxel dose could be reduced to 60 mg/m^2^ to reduce toxic effects.	Progression-free survival, qualityof life, safety. Tolerance
	docetaxel	733 (488/245)	60 (20-84)				
I.Sekine 2009 ^[19]^	gefitinib	245	—	Single-center	—	Gefitinib 250 mg/d was administered orally; docetaxel was administered every 3 weeks as a 1-hour intravenous infusion of 60 mg/m^2^. Patients received treatment until disease progression, intolerable toxicity, or discontinuation for another reason.	ORR, Progression-free survival, DCR, safety. Tolerance
	docetaxel	244	—				
ZHANG Yi 2009^[20]^^b^	gefitinib	26(12/714)	66(34-84)	Single-center	China	Gefitinib 250 mg/d was administered orally; docetaxel was administered as a 1-hour intravenous infusion of 60 mg/m^2^. Patients received treatment until disease progression, intolerable toxicity, or discontinuation for another reason.	tumor response, neutropenia, rash/acne. Neurotoxicity, anaemia
	docetaxel	28(20/78)	61 (40-79)				
LI Hongmei 2010^[21]^	gefitinib	50 (30/ 20)	50.7	Single-center	China	Patients received either 250 mg/d gefitinib orally or 75 mg/m^2^ docetaxel infusion on day 1 every 3 weeks. Patients received treatment with gefitinib or docetaxel until disease progression, unacceptable toxicity, or patient’s withdrawal of consent, and for docetaxel, only the maximum administration of 6 cycles was reached.	survival time, time for tumor progress, qualityof life
	docetaxel	48 (29/19)	48.2				
Dae Ho Lee 2010^[22]^	gefitinib	82 (55/27)	57 (21-74)	Multi-center (6)	Korea	Patients received either 250 mg/d gefitinib orally or 75 mg/m^2^ docetaxel as a 1-hour i.v. infusion on day 1 every 3 weeks. Patients received treatment with gefitinib or docetaxel until disease progression, unacceptable toxicity, or patient's withdrawal of consent, and for docetaxel, only the maximum administration of 6 cycles was reached.	Progression-free survival, tumor responses, survival rate, qualityoflife
	docetaxel	79(45/3479	58(20-73)				
SHANG Shuheng 2009^[23]^	gefitinib	25(15/710)	54(37-71)	Single-center	China	efitinib：250 mg/d, orally, docetaxel：75 mg/m^2^, intravenously over a 1 h period every 3 weeks.	ORR, 1 year survival rate, toxicity
	docetaxel	25(14/711)	52 (39-70)				
Tetsuya Mitsudoml 2010^[24]^	gefitinib	86(27/59)	64.0 (34-74)	Multi-center (36)	Japan	Patients were randomly assigned to receive gefitinib (250 mg/d, orally), or docetaxel (60 mg/m^2^, intravenously over a 1 h period)followed by cisplatin (80 mg/m^2^, intravenously over a 90-min period), with adequate hydration, in cycles of once every 21 days for three to six cydes.Treatment continued until progression of the disease, development of unacceptable toxic effects, a request by the patient to discontinue treatment, serious non-compliance with the protocol, or completion of three to six chemotherapy cycles.	Progression-free survival, tumor responses, survival rate, safety
	cisplatin+docetaxel	86(26/60)	64.0 (41-75)				
ZHANGYuhui 2009^[25]^	gefitinib	35 (12//23)	58(42-78)	Single-center	China	Patients received either 250 mg/d gefitinib orally or Pemetrexed 500 mg/m^2^ docetaxel intravenously with a 10 min period every 3 weeks.	tumorresponses, nonhematologictoxicity, diarrhea
	Pemetrexed	32 (15/17)	56(41-76)				
a: gefitinib group: 26, docetaxel group: 25; b: mean age (range).							

检索Cochrane图书馆、PubMed、CNKI等数据库，共检索到文献2, 139条，排除动物试验、非随机对照试验、Ⅰ期、Ⅱ期临床试验等，最终纳入13个随机对照试验，共6, 207例病例。其中3个研究^[14-16]^为吉非替尼*vs*安慰剂，共3, 110例病例；8个研究^[12, 17-23]^为吉非替尼*vs*多西紫杉醇，共2, 858例病例；1个研究^[24]^为吉非替尼*vs*顺铂+多西紫杉醇，共172例病例；1个研究^[25]^为吉非替尼*vs*培美曲赛，共67例病例。

### 2.2 方法学质量评价结果（表 2）

**2:** 纳入研究的方法学质量 Methodology quality of included studies

Study	Randomization	Allocation concealment	Blinding	Incompleteness of data	Selective outcome reporting	Othersourcesof bias
Giuseppe Giaccone 2004^[14]^	Unclear	Unclear	Double-blind	Yes	Unclear	Unclear
Roy S. Herbst 2004^[15]^	Unclear	Unclear	Double-blind	Yes	Unclear	Unclear
NickThatcher 2005^[16]^	Yes	Yes	Double-blind	Yes	Unclear	Unclear
XIONG Huihua 2008^[17]^	Unclear	Unclear	Unclear	No	Unclear	Unclear
Riichiroh Maruyama 2008^[12]^	Yes	Unclear	Unclear	Yes	Unclear	Unclear
Edwards Kim 2008^[18]^	Yes	Yes	Unclear	Yes	Unclear	Unclear
I.Sekine 2009^[19]^	Unclear	Unclear	Unclear	Yes	Unclear	Unclear
ZHANG Yi 2009^[20]^	Unclear	Unclear	Unclear	No	Unclear	Unclear
LI Hongmei 2010^[21]^	Unclear	Unclear	Unclear	Yes	Unclear	Unclear
DaeHoLee 2010^[22]^	Yes	Unclear	Unclear	Yes	Unclear	Unclear
SHANGShuheng 2009^[23]^	Unclear	Unclear	Unclear	No	Unclear	Unclear
Tetsuya Mitsudomi 2010^[24]^	Yes	Yes	Unclear	Yes	Unclear	Unclear
ZHANG Yuhui 2009^[25]^	Unclear	Unclear	Unclear	Yes	Unclear	Unclear

纳入的13个随机对照试验中，5个研究^[12, 16, 18, 22, 24]^采用了最小化随机、分层随机、中央随机等充分而正确的随机方法，3个研究^[16, 18, 24]^采用了中央电话、传真等正确的隐蔽分组方法，3个研究^[14-16]^报告使用了双盲，但未报告具体的施盲对象。

### 2.3 *Meta*分析结果（表 3）

**3:** *Meta*分析结果 Outcome of *meta* analysis

Endpoint	Intervention	Participant	Heterogeneity		RR	95%CI	*P*
			*P*	*I^2^*			
1 year survival rate	gefitinib *vs* placebo	1, 726/1, 173	0.03	72%	1.05	0.86-1.30	0.62
	gefitinib *vs* docetaxel	1, 078/1, 076	0.94	0%	0.94	0.84-1.04	0.23
CR	gefitinib *vs* placebo	1, 510/1, 048	0.95	0%	1.90	0.87-4.11	0.11
PR	gefitinib *vs* placebo	1, 295/804	< 0.000, 001	95%	2.46	0.35-17.47	0.37
	gefitinib *vs* docetaxel	126/126	0.93	0%	1.22	0.75-1.99	0.43
	gefitinib *vs* Pemetrexed	35/32	-	-	1.14	0.52-2.53	0.74
SD	gefitinib *vs* placebo	959/480	-	-	1.03	0.87-1.12	0.74
	gefitinib *vs* docetaxel	100/98	0.95	0%	0.98	0.67-1.44	0.92
	gefitinib *vs* Pemetrexed	35/32	-	-	1.14	0.63-2.06	0.66
ORR	gefitinib *vs* placebo	1, 510/1, 048	< 0.000, 1	90%	1.61	0.89-2.90	0.11
	gefitinib *vs* docetaxel	1, 067/1, 050	0.25	24%	1.41	1.10-1.80	0.006
	gefitinib *vs* cisplatin+docetaxel	58/59	-	-	1.93	1.26-2.94	0.002
	gefitinib *vs* Pemetrexed	35/32	-	-	1.14	0.52-2.53	0.74
DCR	gefitinib *vs* placebo	959/480	-	-	0.96	0.91-1.02	0.16
	gefitinib *vs* docetaxel	300/289	0.99	0%	1.06	0.88-1.27	0.55
	gefitinib *vs* cisplatin+docetaxel	58/59	-	-	1.19	1.03-1.39	0.02
	gefitinib *vs* Pemetrexed	35/32	-	-	1.14	0.81-1.61	0.44
Total FACT-Limprove rate	gefitinib *vs* placebo	1, 129/563	-	-	1.42	1.16-1.74	0.000, 7
	gefitinib *vs* docetaxel	1, 002/961	0.56	0%	1.66	1.39-1.97	< 0.000, 01
TOI improve rate	gefitinib *vs* docetaxel	928/888	0.21	34%	2.08	1.63-2.65	< 0.000, 01
LCS improve rate	gefitinib *vs* docetaxel	1, 008/964	0.94	0%	1.16	0.98-1.37	0.09
Diarrhea	gefitinib *vs* placebo	1, 830/1, 258	0.03	73%	2.25	1.96-2.60	< 0.000, 01
	gefitinib *vs* docetaxel	1, 105/1, 080	0.19	35%	1.52	1.34-1.73	< 0.000, 01
	gefitinib *vs* cisplatin+docetaxel	87/88	-	-	1.36	0.98-1.87	0.06
	gefitinib *vs* Pemetrexed	35/32	-	-	6.86	1.70-27.68	0.007
Neutropenia	gefitinib *vs* placebo	708/696	0.11	60%	0.98	0.51-1.86	0.94
	gefitinib *vs* docetaxel	1, 025/1, 007	0.000, 2	85%	0.23	0.11-0.49	0.000, 2
	gefitinib *vs* cisplatin+docetaxel	87/88	-	-	0.09	0.04-0.18	< 0.000, 01
	gefitinib *vs* Pemetrexed	35/32	-	-	0.04	0.00-0.60	0.02
Anaemia	gefitinib *vs* placebo	704/696	0.008	86%	1.22	0.32-4.62	0.77
	gefitinib *vs* docetaxel	780/768	0.17	43%	0.29	0.12-0.70	0.006
	gefitinib *vs* cisplatin+docetaxel	87/88	-	-	0.42	0.32-0.56	< 0.000, 01

#### 2.3.1 生存情况

##### 2.3.1.1 中位生存时间

吉非替尼*vs*安慰剂组：2个研究^[14, 15]^报道了中位生存时间，结果显示差异无统计学意义（9.9个月*vs* 10.9个月，*P*=0.456；9.8个月*vs* 9.9个月，*P*=0.638, 5）。吉非替尼*vs*多西紫杉醇组：5个研究^[12, 17, 18, 20, 21]^报道了中位生存时间，其中研究^[17, 18, 20, 21]^的结果分别显示差异无统计学意义（6.1个月*vs* 6.5个月，*P* > 0.05；7.7个月*vs* 8个月，*P* > 0.05；7.1个月*vs* 6.9个月，*P* > 0.05；15.8个月*vs* 16.3个月，*P*=0.48）；研究^[12]^的结果显示差异有统计学意义（11.5个月*vs* 14个月，*P* < 0.05）。

##### 2.3.1.2 1年生存率

文献[14-16]和文献[12, 17, 18, 21, 23]分别报道了吉非替尼对比安慰剂、多西紫杉醇治疗NSCLC的1年生存率，*meta*分析结果显示差异均无统计学意义（RR=1.05, 95%CI: 0.86-1.30, *P*=0.86, *P*=0.62; RR=0.94, 95%CI: 0.84-1.04, *P*=0.23）。

#### 2.3.2 疾病控制情况

##### 2.3.2.1 完全缓解率

研究^[14-16]^报道了吉非替尼对比安慰剂的完全缓解率，*meta*分析结果显示差异无统计学意义（RR=1.90, 95%CI: 0.87-4.11, *P*=0.11）。文献[17, 20, 21, 23]和文献[25]分别报道了吉非替尼对比多西紫杉醇和培美曲赛的完全缓解率，但无1例达到完全缓解。

##### 2.3.2.2 部分缓解率

文献[14, 16]、文献[17, 20, 21, 23]和文献[25]分别报道了吉非替尼对比安慰剂、多西紫杉醇、培美曲赛的部分缓解率，*meta*分析结果显示差异均无统计学意义（RR=2.46, 95%CI: 0.35-17.47, *P*=0.37; RR=1.22, 95%CI: 0.75-1.99, *P*= 0.43; RR=1.14, 95%CI: 0.52- 2.53, *P*=0.74）。

##### 2.3.2.3 疾病无进展率

文献[16]、文献[17, 21, 23]和文献[25]分别报道了吉非替尼对比安慰剂、多西紫杉醇、培美曲赛的疾病无进展率，*meta*分析结果显示差异均无统计学意义（RR=1.03, 95%CI: 0.87-1.12, *P*=0.74; RR=0.98, 95%CI: 0.67-1.44, *P*=0.92; RR=1.14, 95%CI: 0.63-2.06, *P*=0.66）。

##### 2.3.2.4 总有效率

文献[14-16]、文献[25]分别报道了吉非替尼对比安慰剂和培美曲赛的总有效率，*meta*分析结果显示差异均无统计学意义（RR=1.61, 95%: 0.89-2.90, *P*=0.11; RR=1.14, 95%CI: 0.52-2.53, *P*=0.74）。文献[12, 17, 18, 20-23]、文献[24]报道了吉非替尼对比多西紫杉醇、顺铂+多西紫杉醇的总有效率，*meta*分析结果显示差异均有统计学意义（RR=1.41, 95%CI: 1.10-1.80, *P*=0.006; RR=1.93, 95%CI: 1.26-2.94, *P*=0.002）。

##### 2.3.2.5 疾病控制率

文献[16]、文献[12, 20, 21, 23]和文献[25]分别报道了吉非替尼对比安慰剂、多西紫杉醇、培美曲赛的疾病控制率，*meta*分析结果显示差异无统计学意义（RR=0.96, 95%CI: 0.91-1.02, *P*=0.16; RR=1.06, 95%CI: 0.88-1.27, *P*=0.55; RR=1.14, 95%CI: 0.81-1.61, *P*=0.44)。研究^[24]^报道了吉非替尼对比顺铂+多西紫杉醇的疾病控制率，*meta*分析结果显示差异有统计学意义（RR=1.19, 95%CI: 1.03-1.39, *P*=0.000, 7, *P*=0.02）。

#### 2.3.3 生活质量

##### 2.3.3.1 总FACT-L改善率

文献[16]、文献[12, 17-19, 21, 22]报道了吉非替尼对比安慰剂、多西紫杉醇治疗NSCLC的总FACT-L改善率，*meta*分析结果显示差异均有统计学意义（RR=1.42, 95%CI: 1.16-1.74, *P*=0.000, 7; RR=1.66, 95%CI: 1.39-1.97, *P* < 0.000, 01）。

##### 2.3.3.2 TOI改善率

研究^[12, 17-19, 22]^报道了吉非替尼对比多西紫杉醇治疗NSCLC的TOI改善率，*meta*分析结果显示差异有统计学意义（RR=2.08, 95%CI: 1.63-2.65, *P* < 0.000, 01）。

##### 2.3.3.3 LCS改善率

研究^[12, 17, 18, 21]^报道了吉非替尼对比多西紫杉醇治疗NSCLC的LCS改善率，*meta*分析结果显示差异无统计学意义（RR=1.16, 95%CI: 0.98-1.37, *P*=0.09）。

#### 2.3.4 毒副反应

##### 2.3.4.1 腹泻发生率

文献[14-16]、文献[12, 17, 18, 22, 23]分别报道了吉非替尼对比安慰剂、多西紫杉醇治疗NSCLC的腹泻发生率，*meta*分析结果显示差异均有统计学意义（RR=2.25, 95%CI: 1.96-2.60, *P* < 0.000, 01; RR=1.52, 95%CI: 1.34-1.73, *P* < 0.000, 01）。研究^[24]^报道了吉非替尼对比顺铂+多西紫杉醇治疗NSCLC的腹泻发生率，*meta*分析结果显示差异无统计学意义（RR=1.36, 95%CI: 0.98-1.87, *P*=0.06）。

##### 2.3.4.2 中性粒细胞减少发生率

研究^[14, 15]^报道了吉非替尼对比安慰剂治疗NSCLC的中性粒细胞减少发生率，*meta*分析结果显示差异无统计学意义（RR=0.98, 95%CI: 0.51-1.86, *P*=0.94）。文献[12, 17, 18, 20]、文献[24]和文献[25]报道了吉非替尼对比多西紫杉醇、顺铂+多西紫杉醇、培美曲赛治疗NSCLC的中性粒细胞减少发生率，*meta*分析结果显示差异均有统计学意义（RR=0.23, 95%CI: 0.11-0.49, *P*=0.000, 2; RR=0.09, 95%CI: 0.04-0.18, *P* < 0.000, 01; RR=0.04, 95%CI: 0.00-0.60, *P*=0.02）。

##### 2.3.4.3 贫血发生率

研究^[14, 15]^报道了吉非替尼对比安慰剂治疗NSCLC的贫血发生率，*meta*分析结果显示差异无统计学意义（RR=1.22, 95%CI: 0.32-4.62, *P*=0.77）。文献[18, 20, 23]和文献[24]分别报道了吉非替尼对比多西紫杉醇、顺铂+多西紫杉醇治疗NSCLC的贫血发生率，*meta*分析结果显示差异均有统计学意义（RR=0.29, 95%CI: 0.12-0.70, *P*=0.006; RR=0.42, 95%CI: 0.32-0.56, *P* < 0.000, 01）。

## 3 讨论

本研究采用*meta*分析的方法，对国内外吉非替尼治疗NSCLC的随机对照试验的有效性和安全性进行了定量分析。结果显示：吉非替尼相比安慰剂、多西紫杉醇、顺铂+多西紫杉醇、培美曲赛等治疗手段而言，中位生存时间、1年生存率、完全缓解率、部分缓解率、无进展疾病生存率等方面并未显示出优势，但吉非替尼可明显增加化疗患者的总有效率，并提高患者的生存质量，其主要不良反应包括皮疹/痤疮、皮肤干燥、腹泻和贫血等，虽然吉非替尼组的患者贫血和腹泻的发生率略高于其它化疗组，但严重血液毒性的发生率却低于其它化疗组且可以减少疲劳的发生率。

纳入的13个随机对照试验中，仅5个研究^[12, 16, 18, 22, 24]^采用了充分的随机方法，其中1个研究^[16]^采用了最小化随机法，2个研究^[12, 22]^采用了分层随机法，2个研究^[18, 24]^的随机序号在试验中心产生。3个研究^[16, 18, 24]^采用了正确的分配隐藏方法，分别是试验中心电话^[16, 18]^和试验中心传真^[24]^。有研究显示即使用了正确的随机方法，但如果未对随机序列进行有效隐藏，依然会在纳入患者时产生选择性偏倚，影响研究结果的真实性。本研究中大部分指标，如完全缓解、部分缓解、生活质量改善、皮疹/痤疮、疲劳等均为主观测量指标，其真实的测量结果依赖于该指标测量人员是否实施正确的盲法，以降低或避免由此造成的实施和测量偏倚。但纳入的13个随机对照试验中，仅3个研究^[14-16]^明确说明在试验过程中使用了双盲法，且均未告知具体的施盲对象。因此，不排除其存在一定的实施和测量偏倚。此外，除干预措施外的基础药物治疗以及药物剂量在各研究之间存在一定的差异，均可能对该研究结果产生一定的影响。

虽已经有部分临床研究^[16, 18]^显示吉非替尼对亚洲人、非吸烟者、女性和腺癌有较好的疗效，但由于纳入研究的局限性，未能对性别、人种、是否吸烟/癌症的病理分型以及不同剂量等进行亚组分析，有待于今后的研究来进一步完善。除此之外，有研究提出吉非替尼每天剂量为500 mg时，能够改善病人的症状，但是会产生更多的不良反应^[9, 10]^，我们接下来将对500 mg/d的吉非替尼治疗NSCLC的有效性和安全性进行系统的评价。

综上所述，相比较于其它化疗药物，吉非替尼在治疗NSCLC方面具有较好的优势，可作为NSCLC化疗的常规药物，但由于纳入研究的局限性，尚需要高质量的随机对照试验进一步验证其最佳的用药剂量及长期的疗效和安全性。
